# Care for deficits in activities of daily living (ADL) and instrumental ADL and unmet care needs among Austrians aged 65 and over

**DOI:** 10.1007/s10354-026-01133-y

**Published:** 2026-02-24

**Authors:** Thomas Ernst Dorner, Katharina Viktoria Stein, Christoph Gisinger

**Affiliations:** 1 Academy for Ageing Research, “Haus der Barmherzigkeit”, Vienna, Austria; 2 Karl-Landsteiner Institute for Health Promotion Research, Kirchstetten, Austria; 3 Centre for Public Health, Department of Social and Preventive Medicine, Medical University Vienna, Kinderspitalgasse 15/I, 1090 Vienna, Austria

**Keywords:** Gerontology, Disabilities, Functioning, Predictors, Public health, Gerontologie, Behinderungen, Funktionsfähigkeit, Prädiktoren, Öffentliches Gesundheitswesen

## Abstract

This study investigates sociodemographic and health-related factors associated with receiving informal and professional care, as well as unmet care needs, among adults aged 65+ in Austria, using data from the 2019 Austrian Health Interview Survey (*n* = 3408). Overall, 22.2% reported difficulties with at least one ADL and 43.8% with at least one IADL. Among those affected, 72.6% (ADL) and 76.2% (IADL) received help. Technical aids were reported by 35.2% with ADL and 10.6% with IADL limitations. Help from family or friends was common (58.6% ADL; 68.5% IADL), while professional support was less frequent (29.3% ADL; 19.2% IADL). Still, 27.0% of those with ADL and 18.7% with IADL deficits reported unmet care needs. Key predictors for unmet needs included older age, living alone, and chronic conditions such as depression, stroke, incontinence, and inflammatory bowel disease. These findings underscore the importance of tailored interventions to improve care access and support for older adults with functional limitations.

## Abstract (de)

In der vorliegenden Studie wurden soziodemografische gesundheitsbezogene Faktoren untersucht, die mit dem Erhalt informeller und professioneller Pflege einhergehen, sowie unerfüllte Versorgungsbedürfnisse bei Erwachsenen im Alter von 65 Jahren und älter in Österreich. Dazu wurden Daten der Österreichischen Gesundheitsbefragung 2019 verwendet (*n* = 3408). Dabei gaben 22,2 % der Befragten Schwierigkeiten mit mindestens einer Aktivität des täglichen Lebens (ADL) an und 43,8 % mit mindestens einer instrumentellen ADL (IADL). Unter den Betroffenen erhielten 72,6 % (ADL) bzw. 76,2 % (IADL) Hilfe. So wurde technische Unterstützung von 35,2% Personen mit Einschränkungen der ADL und von 10,6% mit Einschränkungen der IADL angegeben. Hilfe von Familienangehörigen oder Freunden war verbreitet (58,6 % ADL; 68,5 % IADL), während professionelle Unterstützung weniger häufig war (29,3 % ADL; 19,2 % IADL). Dennoch berichteten 27,0 % der Personen mit Einschränkungen der ADL und 18,7 % mit Einschränkungen der IADL über unerfüllte Versorgungsbedürfnisse. Zu den entscheidenden Prädiktoren für unerfüllte Bedürfnisse gehörten höheres Alter, das Alleinleben und chronische Erkrankungen wie Depression, Schlaganfall, Inkontinenz und chronisch-entzündliche Darmerkrankungen. Diese Ergebnisse unterstreichen die Bedeutung individuell abgestimmter Maßnahmen, um den Zugang zu Pflege und Unterstützung für ältere Erwachsene mit funktionellen Einschränkungen zu verbessern.

## Introduction

As populations across the world continue to age, understanding the health and care needs of older adults has become an essential area of research [1]. In this context, unmet care needs are a growing concern [2]. Older individuals often face physical and cognitive challenges that can limit their ability to carry out essential activities of daily living (ADLs) and instrumental activities of daily living (IADLs), which lead to a higher need for care [3]. Sociodemographic factors (gender, age, education, country of birth, place of residence, and partnership status), health-related conditions (e.g., chronic diseases, physical ailments like osteoarthritis, back or neck pain, chronic obstructive pulmonary disease, and mental health conditions such as depression), and lifestyle factors (e.g., low physical activity and poor nutrition) are strongly associated with an increased risk of ADL and IADL limitations [4–6].

Coping with deficits in ADLs and IADLs involves a range of strategies aimed at healthy longevity and maintaining independence for as long as possible and delaying or preventing the need for institutional long-term care. Typically, the process of mitigating (I)ADL deficits begins with self-initiated adaptations and the use of assistive (often technical) devices, followed by informal support from lay caregivers, and may eventually extend to professional care services, including therapeutic and social assistance provided in the home. These three levels of extramural care are frequently experienced in a sequential, cascade-like progression, although overlaps between stages are common [7]. As limitations in ADLs and IADLs accumulate, they increasingly shape the type and intensity of required care across all levels—highlighting the critical importance of early interventions aimed at preventing or mitigating care dependency [7]. While the “World Report on Aging and Health” by the Word Health Organization [8] provides important international insights into aging and care needs, Austria-specific data are essential to understand the prevalence of ADL and IADL limitations, care receipt, and unmet care needs within the Austrian healthcare and social system.

Informal care, typically provided by family members or friends, is often the first line of support for individuals with care needs. However, informal care may be inconsistent, especially in the context of complex or long-term health conditions. The burden placed on informal caregivers can result in stress, burnout, and a diminished capacity to provide adequate support, all of which can compromise the overall quality of care [9]. Professional care, by contrast, is generally delivered by healthcare and social service providers and includes services such as home nursing, physiotherapy, medical consultations, and formal social assistance (e.g., home help services, meals-on-wheels, and day care programs). In any case, geriatric care in the extramural setting requires a person-centered approach, putting focus on the whole person with all individual resources and needs, and an inter-professional cooperation with inclusion of the relatives [10].

Deficits in self-care, informal care, and professional care may lead to unmet care needs—situations in which individuals do not receive the assistance they require [11]. These unmet needs can have severe consequences, including deteriorating physical and mental health, loss of independence, and reduced quality of life. Understanding and addressing unmet care needs is critical, as they not only affect the well-being of older individuals but also place a greater strain on health and social care systems when conditions worsen due to inadequate early support [12–14].

A range of sociodemographic factors, such as age, sex, educational attainment, household size, and marital status, have been shown to influence the likelihood of receiving both informal and professional care, in some cases they are inversely associated with informal and professional care. In addition, health-related variables, including the type and severity of chronic illnesses, functional limitations, and mental health conditions, also shape the nature and extent of care received [15, 16].

Gender in particular plays a significant role in shaping care dynamics for individuals with ADL and IADL limitations [17]. This is partly due to differences in life expectancy—women are more likely to live longer and therefore more often reach an age where care needs increase, often without a partner present. In addition, traditional gender roles continue to influence patterns of care provision and receipt. Women are more likely to be caregivers throughout their lives and may also be more likely to receive informal care in old age due to established social support networks. Conversely, older men may be less accustomed to seeking or accepting help, potentially leading to higher rates of unmet care needs. Gender norms may also affect how care tasks are perceived, with certain types of assistance (e.g., personal hygiene) possibly carrying more stigma for men, resulting in delayed care-seeking [18, 19].

Identifying the factors associated with the receipt of informal and professional care, as well as the prevalence of unmet care needs, is vital for designing effective interventions that support older adults in maintaining their health, well-being, and autonomy. It is also essential for policymakers and healthcare providers, as it helps to ensure that resources are directed toward those most in need of care and support. In this study, we aim to explore the sociodemographic and health-related factors that influence the receipt of informal and professional care, as well as the presence of unmet care needs, among older adults aged 65 years and older in Austria.

## Materials and methods

This study draws on data from the most recent wave of the Austrian Health Interview Survey (ATHIS), conducted in 2019 [20]. The survey was implemented by Statistics Austria, the national statistical office, on behalf of the Austrian Ministry of Health. ATHIS represents Austria’s contribution to the European Health Interview Survey (EHIS), a harmonized instrument regularly conducted across EU member states to collect information on health status, health determinants, healthcare utilization, and socioeconomic characteristics of the population [21, 22]. The survey targets individuals aged 15 years and over who are registered in Austria’s central population register. It is carried out across 32 designated geographical regions, each with a predetermined sample size. To improve response rates, participants received multiple reminders and were offered a gift voucher as an incentive. Following a comprehensive analysis of non-response patterns, missing data were imputed based on sex, age, educational attainment, and region of residence. The 2019 ATHIS employed a mixed-mode data collection approach, combining computer-assisted personal interviewing (CAPI) with a Web-based questionnaire. The final net sample comprised 15,461 individuals, corresponding to a response rate of 50.5%. The response rate was somewhat higher among the older population: 57.8% for those aged 60–74 years and 53.7% for those aged 75 and older.

To assess limitations in ADLs, we employed dimensions adapted from Katz et al. [23], encompassing five items: eating or drinking, getting into/out of bed, dressing/undressing, using the toilet, and bathing or taking a shower. For the assessment of IADLs, we utilized dimensions adapted from Lawton and Brody [24], comprising seven items: preparing meals, using the telephone, shopping, managing medication, undertaking light housework, undertaking heavy housework, and managing money. The corresponding survey question was: “Do you usually have difficulty doing any of the following activities by yourself without help?” Response options included “no difficulty,” “some difficulty,” “a lot of difficulty,” and “cannot do at all/unable to do by myself.” For analytical purposes, the last three responses were grouped together under the category “yes”. If a difficulty was reported in any ADL or IADL domain, a follow-up question was posed—separately for the ADL and IADL sections—asking: “Do you usually have help with at least one of these activities?” In cases where the response was “yes,” three additional questions were presented: “Is this help a technical aid?”; “Is this help from friends/family members?”; and “Is this help from professional nursing or support staff?” Respondents were also asked: “Do you need more help with at least one of the activities mentioned?”

The following sociodemographic variables were included in the analysis: sex (male, female); age (recorded in 5‑year intervals and categorized into 65–79 years and 80 years and older); highest level of education (primary education = completion of compulsory schooling by age 15; secondary education = apprenticeship, vocational training, or completion of upper secondary school; tertiary education = attendance at a university or university of applied sciences); country of birth (Austria vs. other countries); and degree of urbanization (residing in Vienna, the country’s only metropolis with a population of approximately 2 million, vs. other federal provinces). Marital or relationship status was categorized as either married/living in a relationship or other, and household size was classified as living alone, living with one other person, or living in a household of three or more persons.

The presence of chronic conditions was assessed through the question: “Have you had any of the following illnesses or health problems in the last 12 months?” followed by a list of 19 specific chronic conditions. For the purposes of this study, only those chronic illnesses with a strong association with limitations in ADLs or IADLs were included in the analysis. In selected cases, namely, osteoarthritis, incontinence, depression, and chronic inflammatory bowel disease, respondents were additionally asked: “Was the diagnosis made by a doctor?” For these conditions, only self-reported doctor-diagnosed cases were considered in the analysis.

For the present analysis, we included only individuals aged 65 years and older, resulting in a total sample of 3408 participants. All analyses were conducted using weighted data, with sociodemographic characteristics applied as weighting factors. Descriptive statistics were used to summarize the data, and proportions were reported accordingly. Furthermore, binary logistic regression analyses were carried out to examine associations with three dependent variables: receiving informal care, receiving professional care, and experiencing unmet care needs. Sociodemographic and health-related variables served as independent variables in these models. All logistic regression models were mutually adjusted for the included covariates. Results are presented as odds ratios (ORs) with corresponding 95% confidence intervals (95% CIs). The coefficient of determination (*R*^2^) is reported as an indicator of model fit.

The secondary analysis of the ATHIS-19 database was approved by the Ethics Committee of the Medical University of Vienna (EK #1263/2021).

## Results

The sociodemographic and health-related characteristics of individuals aged 65 years and older included in the survey are presented in Table 1. The most commonly reported chronic condition in this population was chronic back or neck pain, followed by osteoarthritis. Approximately 10% of participants reported having coronary heart disease or a history of myocardial infarction, urinary incontinence, chronic obstructive pulmonary disease (COPD), or depression.

**Table 1:** Sociodemographic and health-related characteristics of the 3408 participants aged 65 years and older of the Austrian Health Interview Survey 2019

Characteristic	Category	Percentage
Sex	Male	44.3
	Female	55.7
Age	65–79 years	74.4
	≥ 80 years	25.6
Education level	Primary	31.9
	Secondary	59.3
	Tertiary	8.8
Country of birth	Austria	87.7
	Not Austria	12.3
Urbanization	Vienna	18.7
	Other federal states	81.3
Family status	In a relationship	60.5
	Not in a relationship	39.5
Household size	One person	32.9
	Two persons	58.8
	Three persons or more	8.3
Chronic diseases	COPD	9.6
	History of stroke	5.1
	Renal failure	6.4
	Osteoarthritis doctor diagnosed	31.5
	Incontinence doctor diagnosed	11.6
	Depression doctor diagnosed	7.3
	CIBD doctor diagnosed	5.1
	CHD or history of MI	13.0
	Chronic back or neck pain	44.2
Problems with ADL	Eating or drinking	6.6
	Transferring (e.g., from bed to chair)	17.3
	Dressing	16.0
	Toileting	9.6
	Bathing or showering	16.1
Problems with IADL	Preparing meals	14.9
	Using the telephone	9.7
	Doing shopping	20.4
	Managing medication	12.8
	Doing light housework	18.7
	Doing heavy housework	40.3
	Managing financial matters	16.7

*COPD* chronic obstructive pulmonary disease, *CIBD* chronic inflammatory bowel disease, *CHD* coronary heart disease, *MI* myocardial infarction, *ADL* activities of daily living, *IADL* instrumental activities of daily living

Among men, 17.9% reported difficulties with at least one ADL, while the corresponding proportion among women was 25.7%. The most frequently reported ADL limitations were transferring (e.g., getting in or out of bed), bathing or showering, and dressing. Regarding IADLs, 35.1% of men and 50.8% of women reported at least one difficulty. The most prevalent IADL limitation was performing heavy housework, followed by shopping, doing light housework, and managing financial affairs.

Table 2 shows the proportions of men and women with ADL/IADL problems who received help from different sources. About two-thirds of men with ADL/IADL problems received help for at least one ADL/IADL, with even higher corresponding proportions in women. About one-third of men and women with ADL problems had technological aids to help them manage. This proportion was about 10% in men and women with IADL problems. More than half of the men and women with ADL problems received help from family members or friends. This proportion was about two-thirds among men and women with IADL problems. Almost 20% of men and almost twice as many women with ADL problems sought professional help. In people with IADL problems, the proportion who had professional help was lower. Additionally, about one-fifth of men and one-third of women with ADL problems reported that they needed more help. This proportion was about one-fifth among men and women with IADL problems.

**Table 2:** Proportion (%) of men and women aged 65 and over with at least one ADL/IADL problem, by type of assistance received

	People with problems in at least one ADL		People with problems in at least one IADL	
	Men (*N* = 270)	Women (*N* = 487)	Men (*N* = 530)	Women (*N* = 964)
Help with at least one ADL/IADL activity	69.7	74.3	69.2	80.1
Technical aids	32.2	36.9	8.5	11.7
Help from family/friends	58.3	58.7	62.7	71.8
Professional help	18.3	35.4	14.3	22.3
Would need more help	18.9	31.5	17.0	19.7

*ADL* activities of daily living, *IADL* instrumental activities of daily living

Table 3 presents the results of the multivariate logistic regression analyses for receipt of informal care, professional care, and unmet care needs among individuals aged 65 years and older with ADL limitations. A higher likelihood of receiving informal care from family members or friends was associated with older age, lower educational attainment, living in larger households, and having a diagnosis of COPD, a history of stroke, depression, or coronary heart disease. The likelihood of receiving professional care was higher among women, individuals of older age, and those affected by stroke or urinary incontinence. Conversely, individuals with chronic back or neck pain were less likely to receive professional assistance for ADL difficulties. Factors linked to a higher likelihood of unmet care needs for ADL problems included female sex, not being married or in a relationship, and having a history of stroke or incontinence (Table 3).

**Table 3:** Factors associated with informal care, professional care, and unmet care needs in 757 Austrian people aged 65 years and over with deficits in at least one ADL domain

	Informal care		Professional care		Unmet care needs	
	OR	95% CI	OR	95% CI	OR	95% CI
Male Female	1 0.89	– 0.61–1.30	1 2.04	– 1.30–3.22	1 1.93	– 1.27–2.92
65–79 years 80+ years	1 2.46	– 1.72–3.53	1 2.59	– 1.70–3.92	1 1.37	– 0.93–2.00
Primary education Secondary education Tertiary education	4.17 2.29 1	1.72–10.13 0.96–5.49 –	0.53 0.61 1	0.20–1.43 0.23–1.63 –	1.06 1.53 1	0.39–2.86 0.57–4.09 –
Born in Austria Born not Austria	1 0.85	– 0.52–1.37	1 1.44	– 0.83–2.48	1 1.26	– 0.76–2.09
City (Vienna) Other federal states	1 1.38	– 0.93–2.05	1 1.42	– 0.87–2.33	1 0.92	– 0.59–1.41
In a relationship/married Not in a relationship/not married	1 1.18	– 0.65–2.14	1 1.36	– 0.72–2.56	1 2.10	– 1.18–3.72
One-person household Two-person household Three-person or more household	1 2.78 4.10	– 1.55–4.98 2.01–8.37	1 0.61 0.60	– 0.33–1.10 0.31–1.15	1 1.56 1.15	– 0.90–2.69 0.62–2.16
COPD History of stroke Renal failure Doctor-diagnosed osteoarthritis Doctor-diagnosed incontinence Doctor-diagnosed depression Doctor-diagnosed CIBD CHD or history of MI Chronic back or neck pain	1.84 1.70 1.36 0.88 1.36 1.73 0.98 1.75 1.07	1.10–3.08 1.00–2.89 0.84–2.20 0.62–1.25 0.94–1.97 1.09–2.74 0.54–1.78 1.15–2.67 0.75–1.54	0.66 2.54 1.44 0.74 3.51 0.66 0.50 1.25 0.52	0.37–1.18 1.51–4.28 0.86–2.40 0.49–1.10 2.37–5.20 0.40–1.08 0.24–1.03 0.79–1.96 0.35–0.78	1.47 1.95 1.23 0.71 1.56 0.94 1.03 1.18 1.14	0.91–2.38 1.21–3.15 0.76–1.97 0.49–1.04 1.07–2.27 0.60–1.49 0.56–1.89 0.78–1.79 0.77–1.69
Nagelkerke’s *R*^2^	0.226		0.305		0.113	

Result of a binary logistic regression analysis, all variables are mutually adjusted for each other. Results are shown in OR and 95% CI

*ADL* activities of daily living, *OR *odds ratio,* COPD* chronic obstructive pulmonary disease, *CIBD* chronic inflammatory bowel disease, *CHD* coronary heart disease, *MI* myocardial infarction

According to the multivariate logistic regression models shown in Table 4, the probability of receiving informal care among those with IADL limitations was greater in women, older individuals, those with lower educational attainment, individuals living in households of three or more people, and those diagnosed with depression, chronic inflammatory bowel disease, or coronary heart disease. Factors associated with receiving professional care in this group included older age, higher educational attainment, urban residence, not being married or in a relationship, and having a diagnosis of renal failure or urinary incontinence. By contrast, chronic inflammatory bowel disease and chronic back or neck pain were associated with a reduced likelihood of receiving professional care. Unmet care needs among individuals with IADL limitations were more common in those of older age and in those diagnosed with depression or chronic inflammatory bowel disease.

**Table 4:** Factors associated with informal care, professional care, and unmet care needs in 1494 Austrian people aged 65 years and over with deficits in at least one IADL domain

	Informal care		Professional care		Unmet care needs	
	OR	95% CI	OR	95% CI	OR	95% CI
Male Female	1 1.42	– 1.10–1.83	1 1.38	– 0.98–1.93	1 0.97	– 0.71–1.33
65–79 years 80+ years	1 1.89	– 1.47–2.43	1 2.32	– 1.72–3.14	1 1.76	– 1.32–2.36
Primary education Secondary education Tertiary education	3.43 2.50 1	2.05–5.73 1.52–4.11 –	0.37 0.47 1	0.20–0.67 0.26–0.85 –	0.89 0.76 1	0.48–1.63 0.42–1.40 –
Born in Austria Born not Austria	1 1.23	– 0.86–1.75	1 0.68	– 0.43–1.06	1 1.08	– 0.73–1.61
City (Vienna) Other federal states	1 0.81	– 0.60–1.08	1 0.67	– 0.48–0.93	1 0.76	– 0.55–1.05
In a relationship/married Not in a relationship/not married	1 1.02	– 0.64–1.63	1 2.15	– 1.28–3.61	1 1.60	– 0.98–2.60
One-person household Two-person household Three-person or more household	1 1.41 2.89	– 0.88–2.28 1.63–5.14	1 0.79 0.70	– 0.48–1.28 0.40–1.24	1 1.22 0.88	– 0.75–1.96 0.51–1.53
COPD History of stroke Renal failure Doctor-diagnosed osteoarthritis Doctor-diagnosed incontinence Doctor-diagnosed depression Doctor-diagnosed CIBD CHD or history of MI Chronic back or neck pain	0.71 1.13 1.00 0.92 1.32 1.76 1.85 1.42 1.20	0.50–1.00 0.75–1.70 0.67–1.47 0.72–1.18 0.96–1.80 1.19–2.60 1.13–3.02 1.04–1.94 0.94–1.53	0.75 1.41 1.56 1.16 2.29 1.11 0.47 0.98 0.67	0.48–1.17 0.91–2.21 1.01–2.42 0.85–1.57 1.67–3.14 0.73–1.66 0.25–0.87 0.69–1.40 0.50–0.91	1.06 1.33 1.11 1.14 1.30 1.66 1.71 1.18 1.07	0.72–1.56 0.87–2.04 0.73–1.68 0.85–1.53 0.95–1.78 1.14–2.42 1.09–2.68 0.85–1.64 0.80–1.44
Nagelkerke’s *R*^2^	0.114		0.195		0.074	

Result of a binary logistic regression analysis, all variables are mutually adjusted for each other. Results are shown in OR and 95% CI

*IADL* instrumental activities of daily living, *OR *odds ratio,* COPD* chronic obstructive pulmonary disease, *CIBD* chronic inflammatory bowel disease, *CHD* coronary heart disease, *MI* myocardial infarction

## Discussion

This study examined the sociodemographic and health-related factors associated with the receipt of informal care, professional care, and unmet care needs in individuals aged 65 years and older with limitations in activities of daily living (ADL) and instrumental activities of daily living (IADL) in Austria. Our findings identify several sociodemographic and health-related factors that significantly influence the likelihood of receiving different types of care and the presence of unmet care needs in this age group, which is in line with the literature [25–27].

It is encouraging to observe that the vast majority of individuals with ADL and IADL limitations receive some form of assistance. Technical aids appear to play a substantial role in supporting individuals with ADL difficulties, whereas they are seldom utilized for IADL limitations. Although the specific types of assistive devices were not captured in the data, it is likely that these include items such as grab rails, mobility aids (e.g., walking frames, sticks), bathing or toileting equipment, and devices designed to support dressing or eating. Informal support from family members or friends was reported by approximately two-thirds of individuals, particularly in relation to IADL limitations, emphasizing the central role of informal caregiving. By contrast, professional assistance was accessed by significantly fewer individuals, although notably, more women than men received such support. This finding highlights that the majority of care for both ADL and IADL needs is provided by laypersons rather than professionals. This reliance on informal care may present challenges in the future, particularly in light of demographic changes and increasing work-related demands. As people have children later in life, many may find themselves in the so-called sandwich generation, facing the dual responsibility of caring both for children and for aging parents, which may limit their ability to provide informal care [28]. While fewer than 20% of individuals with ADL or IADL limitations reported unmet care needs, this figure rises to over 30% among women with IADL limitations. Although these proportions may not initially appear high, unmet care needs could result in substantial challenges in the coming years.

In adjusted analyses, female sex was associated with a higher likelihood of receiving professional care and experiencing unmet care needs among individuals with ADL limitations, and a higher likelihood of receiving informal care among individuals with IADL limitations. Age and marital status were partially controlled for in this analysis, suggesting that the observed effects may not be attributed to these variables. However, it is possible that the difficulties associated with ADL and IADL limitations are more pronounced in women due to differences in hormones (especially sex hormones), and as a consequence to a higher likelihood of sarcopenia and frailty [29], necessitating greater support with ADL and IADL deficits compared to men.

Advanced age was associated with (1) a higher likelihood of receiving both informal and professional care among individuals with ADL and IADL deficits and with (2) unmet care needs in those with IADL deficits. Older adults are more likely to require both informal and professional care due to known risk factors for ADL and IADL limitations, including low physical activity, sarcopenia, malnutrition, frailty, and the greater functional impairments experienced by older age groups [30, 31].

Lower educational attainment was associated with a very high likelihood of receiving informal care from relatives or friends and a very low likelihood of receiving professional care, particularly for individuals with IADL deficits. One explanation for this could be that higher education is associated with greater health literacy and higher income, factors that increase the ability to organize and afford professional care. And higher socioeconomic status in older adults is well known to be associated with overall better healthcare access [32]. The low probability of informal care among individuals with higher education may reflect the fact that they are more likely to already receive professional care.

Living in a large city was associated with a higher likelihood of receiving professional care among individuals with IADL deficits. The urban–rural divide in access to professional care is well established, with urban residents typically having better access to healthcare services [33].

Not being married was associated with a higher likelihood of unmet care needs among individuals with ADL deficits and with receiving professional care among individuals with IADL deficits. A higher household size was clearly associated with receiving informal care, which is not surprising, as individuals living in partnerships or larger households may have more social support from family members [25]. Living alone or lacking a partner may contribute to increased care needs, particularly when individuals face chronic health conditions.

Medical diagnoses were associated with care receipt and unmet care needs in different ways. Chronic conditions such as a history of stroke, coronary heart disease, depression, chronic inflammatory bowel disease, or COPD were associated with a higher likelihood of receiving informal care, with some also increasing the likelihood of professional care. This underscores the significant impact of multiple health issues on the need for family or professional assistance to manage ADL and IADL limitations. The association between depression and the need for help with ADL and IADL limitations is well documented, as depression often results in diminished motivation and energy, which can impair an individual’s ability to perform routine tasks. Incontinence was significantly associated with professional care (but not informal care), with the highest odds ratios compared to other diagnoses. This suggests that, particularly in cases of incontinence, professional care is sought due to feelings of shame or a desire to avoid burdening family members, or because family members feel overwhelmed by managing the condition themselves. Some conditions, such as chronic back or neck pain (for ADL and IADL deficits) and chronic inflammatory bowel disease (for IADL deficits), were inversely associated with the likelihood of receiving professional care compared to those without these diagnoses. This may reflect either (1) a gap in the availability or accessibility of specialized care for musculoskeletal or gastrointestinal conditions, (2) a greater likelihood of individuals managing these conditions independently or relying on informal care, or (3) the possibility that individuals with these conditions are already in long-term professional care. Finally, unmet care needs were found to be associated with a history of stroke and incontinence among individuals with ADL deficits, and with depression or chronic inflammatory bowel disease among those with IADL deficits. These findings emphasize the critical need for targeted interventions to address the care needs of older adults, particularly those with multiple chronic conditions [34, 35]. While our study focuses on Austria, it should be noted that the relevance of specific medical diagnoses for planning social and healthcare services may vary across countries with different care systems. Future research could also explore how geriatric rehabilitation or prevention programs might help reduce care needs and unmet needs.

While this study provides valuable insights into the factors influencing care receipt and unmet care needs among older adults, several limitations should be considered. First, the cross-sectional design prevents us from drawing conclusions about causality. Longitudinal studies are needed to better understand the temporal relationships between sociodemographic factors, health conditions, and the dynamics of care provision. Additionally, while self-reported data on chronic conditions and care needs is useful, it may be subject to recall bias or inaccuracies in reporting. The use of a modified Lawton Instrumental Activities of Daily Living index represents another limitation of our study. This index, originally developed in the 1960s, inherently reflects traditional gender roles and may thus introduce bias. Moreover, several IADL items have changed substantially since then. For example, “telephone use” today generally means operating a smartphone, which requires more advanced sensory, tactile, and cognitive skills. Similarly, “managing finances” now primarily involves online and internet banking, often including multifactor authentication, adding complexity and new demands on users. The index therefore does not capture these evolving and more complex modern tasks that are increasingly relevant to daily functioning. It would also have been valuable to include standardized assessments of geriatric syndromes such as frailty and sarcopenia; however, the dataset used does not contain such clinical measures. In addition, we used a pragmatic cutoff of at least one ADL or IADL limitation to identify care needs. While this approach ensures sensitivity, it does not capture the heterogeneity of functional impairment. Future research could also examine how the availability of support services contributes to differences in care provision and unmet needs.

## Conclusion

This study underscores the importance of sociodemographic and health-related factors in determining the need for informal and professional care among older adults. Our findings highlight the complexity of care needs in this population, suggesting that interventions aimed at improving access to care should consider a range of factors, including socioeconomic status, health conditions, and the availability of informal support networks. Addressing unmet care needs, particularly for individuals with common medical diagnoses as assessed in this study, will be crucial as the population ages and the demand for healthcare services continues to rise.
